# The Symbiosis of Virtual Reality Exposure Therapy and Telemental Health: A Review

**DOI:** 10.3389/frvir.2022.848066

**Published:** 2022-02-25

**Authors:** Triton Ong, Hattie Wilczewski, Hiral Soni, Quinn Nisbet, Samantha R. Paige, Janelle F. Barrera, Brandon M. Welch, Brian E. Bunnell

**Affiliations:** 1 Doxy.me Research, Doxy.me Inc., Rochester, NY, United States,; 2 Biomedical Informatics Center, Public Health and Sciences, Medical University of South Carolina, Charleston, SC, United States,; 3 Innovation in Mental Health Lab, Department of Psychiatry and Behavioral Neurosciences, University of South Florida, Tampa, FL, United States

**Keywords:** virtual reality, exposure therapy, VRET, telemedicine, telehealth, phobia, anxiety, mental health

## Abstract

Phobias and related anxiety are common and costly mental health disorders. Experts anticipate the prevalence of phobias will increase due to the COVID-19 pandemic. Exposure therapies have been established as effective and reliable treatments for anxiety, including recent innovations in virtual reality-based exposure therapy (VRET). With the recent advent of telemental health (TMH), VRET is poised to become mainstream. The combination of VRET and TMH has the potential to extend provider treatment options and improve patient care experiences. In this narrative review, we describe how recent events have accelerated VRET + TMH, identify barriers to VRET + TMH implementation, and discuss strategies to navigate those barriers.

## INTRODUCTION

Phobias are characterized by the overwhelming fear of an object or situation. About one in 10 adults and one in five adolescents are diagnosed with phobia each year (NIMH) (NIMH Specific Phobia, 2022). Phobias and related anxiety disorders cost $122 billion in lost productivity and $135 billion in health care annually (Konnopka and König, 2020). People living with phobias can experience disadvantages in school, work, and socialization that elevate risks of depression, substance abuse, deteriorating wellbeing, and suicide (Chartier et al., 2003; Wersebe et al., 2018; Salokangas et al., 2019). If untreated, phobias and related anxiety disorders can have serious impacts on the economy, health care systems, and people’s daily lives. Unfortunately, as few as 10% of people with phobias seek professional care, less than half of whom receive successful treatment (Wardenaar et al., 2017; de Vries et al., 2021).

Exposure therapy is the gold standard treatment for phobias and anxiety (Steinman et al., 2016). Using exposure therapy, providers help patients gain control of their fear gradually in controlled settings (Vinograd and Craske, 2020). In-person exposure (often called *in vivo* exposure therapy; IVET) produces faster and larger symptom improvements with lower rates of relapse compared to non-exposure alternatives (Wolitzky-Taylor et al., 2008). However, staging phobic experiences for IVET can be costly and unfeasible in office settings (Becker et al., 2004; Deacon and Farrell, 2013; Meyer et al., 2014). Providers can guide patients to imagine phobias with programmatic instructions instead of IVET (Hecker, 1990), but imaginal exposure is unideal since providers cannot monitor patient imaginations (Markowitz et al., 2015; Berman et al., 2021). These barriers prevent people with phobias from receiving the most effective and highest quality of care.

Virtual reality (VR) is an emerging solution to the limitations of *in vivo* and imaginal exposure therapy. Virtual reality-based exposure therapy (VRET) uses computer simulations to evoke the same responses as real-life phobic experiences (Gramlich et al., 2021). The physical absence of fearful situations helps patients approach, accept, engage, and adhere more with VRET than IVET with equal or better outcomes (Deng et al., 2019; Wechsler et al., 2019; Horigome et al., 2020). VRET simulations can be made to look, sound, and feel real, resulting in strong ecological validity and transfer of treatment effects (Morina et al., 2015). Providers also benefit from VRET with favorable costs, safety, efficiency, and near total control over patient experiences compared to non-VR exposure therapies (Boeldt et al., 2019). Despite its benefits, mental health providers have not yet embraced VRET for widespread use (Rizzo and Koenig, 2017).

Mental health providers are more likely to adopt a technology if it is easy to use and helps improve patient care, as demonstrated recently with telemedicine (Cowan et al., 2019). Before 2020, mental health providers spent less than 25% of their time delivering telehealth services (Glueckauf et al., 2018; Bunnell et al., 2020). Unresponsive insurance coverage made telemental health (TMH) difficult to use (Aboujaoude, 2018; Adams et al., 2018). Upon the onset of COVID-19, emergency public health policies granted acceptance of TMH which has since been used to conduct as much as 85% of mental health services (Pierce et al., 2020; Reilly et al., 2020). Providers and patients now demand these policy changes be made permanent and TMH uptake become the new norm (Whaibeh et al., 2020; Molfenter et al., 2021; Renn et al., 2021). TMH was once niche but has proven to be an essential care option. VRET is positioned to join that fate in symbiosis.

In this new norm of telemedicine, the human touch of in-person care must be adapted for remote services. VRET is effective for a variety of anxiety and stress-related mental health conditions, demand for which is anticipated to rise in the fallout of COVID-19. The union of telemedicine and VRET can expand clinical options today, prepare for the telehealth landscape of tomorrow, and set the groundwork for the next evolutions of mental health care. In this article, we describe how current events set the stage for remote administration of VRET, how the delivery of VRET via TMH (VRET + TMH) can address trends in mental health demands, and the challenges stakeholders must navigate toward the future.

## BACKGROUND AND CONTEXT FOR VRET + TMH

### Mainstream VR Is Here

VR systems could cost as much as $250,000 USD in the 1980s (Sorene, 2014). The advent and development of personal computers produced clinical VR systems around $10,000 USD in the 1990s (North et al., 1997). Now, some of the most advanced VR systems to date are designed for home entertainment and can be purchased for $2,000 USD (Greenwald, 2021). Popular all-in-one VR systems are available around $300 USD (Wickens, 2021). Use of VR for mental health has followed these trends with research using as little as $5 USD of VR equipment (Lindner et al., 2019a; Donker et al., 2019; Patel et al., 2020). Equipment costs are no longer considered a major barrier to VRET (Lindner et al., 2019b), and consumer technology companies are racing to establish mainstream VR adoption. Facebook has dedicated 17% of its workforce to get VR into one billion homes (Hamilton, 2021). Others like Google, Apple, Sony, and Netflix are also investing heavily in consumer VR and AR (Nanou, 2021). Broad access to sophisticated VR is our present reality.

There is much potential for VR in mental health care. Real-time interaction in VR therapy allows patients to feel more comfortable and present with providers (Mishkind et al., 2017; Park et al., 2019). Because VR feels real, skills practiced and acquired in VR transfer well to real life (Opriş et al., 2012; Morina et al., 2015). The realistic experiences enabled by VR can enhance mental health treatments in ways considered unfeasible for in-person or current telemedicine approaches (e.g., re-experiencing combat for PTSD therapy) (Parsons and Rizzo, 2008; Maples-Keller et al., 2017). VR is also engaging with constant, game-like feedback to promote treatment adherence and outcomes (Benbow and Anderson, 2019). As a result, VR allows patients to practice skills both in real-time with their providers and on their own as part of treatment plans (Chan et al., 2018; Wiederhold and Riva, 2019). These benefits can make VR-based therapies more convenient and flexible than in-person care, more realistic and conducive to therapeutic presence than telemedicine, and unique in its ability to provide tailored simulation experiences for mental health treatment.

### We Need VRET + TMH for the Other Pandemic

These have been hard times for mental health. The COVID-19 pandemic began in early 2020 with sudden and global mandates of social distancing, travel restrictions, and sheltering-in-place. Health care workers suffered extreme social and emotional impacts of the pandemic (Du et al., 2020), and this stress soon spread to the general public (Fang et al., 2020; Liu et al., 2021). Frequent changes in public health policy lead to confusion about personal health practices (Singh and Ravinetto, 2020; The Lancet Infectious Diseases, 2020). Sleep quality declined globally (Gupta et al., 2020; Pérez-Carbonell et al., 2020). People stayed inside under a relentless assault of bad news: police brutality and widespread civil unrest (Taylor, 2021), months-long and devastating wildfires (Arjmand et al., 2021), chronic resource shortages and skirmishes over toilet paper (Labad et al., 2021), reports of giant invasive hornets (Higgins, 2020), announcement of unavoidable climate consequences (McGrath, 2021), and mounting evidence that our online social lives have been manipulated with harmful intent (Hatmaker, 2021; Ortutay, 2021). The state of the world, apparently, keeps getting worse.

Experts now warn of a secondary pandemic in the psychological fallout of COVID-19. About one in five people are at risk for anxiety, phobia, and other mental health disorders due to ongoing isolation, world events, and lockdown conditions (Lyons et al., 2020; Cavicchioli et al., 2021). Elevated symptoms of mental distress have been reported by 31–82% of the general population (Xiong et al., 2020; Wu et al., 2021). Pandemic unemployment fluctuates while traditional paths to professional advancement falter (Berkowitz and Basu, 2021), keeping many under threats of job loss and homelessness (Le and Nguyen, 2021; Lin et al., 2021). With nowhere else to be, sheltering-in-place resulted in escalating rates of domestic abuse (Kovler et al., 2021). School closures delayed the academic, physical, and social development of young people for more than a year (Ghosh et al., 2020; Hoffman and Miller, 2020). Prolonged quarantine increased risks for loneliness, depression, and suicide (Kim and Jung, 2021; Pirkis et al., 2021), especially for those at risk before the pandemic (Chatterjee et al., 2020). Global stress remains high as people worry about their own health and the safety of loved ones (Bonsaksen et al., 2021). The ongoing COVID-19 pandemic and its effects are considered an intergenerational trauma upon peoples’ lives and hopes for the future (Bridgland et al., 2021).

At the time of this publication, coordinated health efforts have vaccinated 63% of the US population against COVID-19 (Carlsen, 2021). In many places, mask mandates have been lifted while international travel and public events rebound. However, hopes of a return to pre-pandemic life are threatened by novel COVID variants which may be deadlier, more contagious, and resistant to previous vaccination (CDC, 2021). Global outbreaks led to the enactment of new public health restrictions, which are being met with widespread protests and violence (BBC News, 2021). COVID-related helplessness seem set to grow among the public. Social avoidance, distrust, anxiety around strangers, compulsive sanitation behaviors, and pervasive fear are now common around the world (Heiat et al., 2021; Lindinger-Sternart et al., 2021; Rosenberg Danziger et al., 2021; Samuels et al., 2021). Pandemic-based phobias may be emerging around hospitals and vaccines, life in public spaces, and screen aversion related to distance learning and remote work (Brown, 2021; McMurdock, 2021; Nardi and Cosci, 2021; Willis et al., 2021; Yakobi and Cheng, 2021). Research, development, and deployment of VRET + TMH will be a critical need for current and future mental health (Shah et al., 2020; Wanling Zhang et al., 2020).

## BENEFITS OF VRET + TMH

Telemedicine has proven to reduce wait times, travel burdens, and the overall stress of patient health care experiences (Lehoux et al., 2000; Gajarawala and Pelkowski, 2021). Mental health services benefit particularly from remote care, allowing patients to seek treatment that feels more private and less stigmatized (Knaak et al., 2017; Goldkind and Wolf, 2021; Renn et al., 2021). However, TMH still leaves some unsatisfied. Patients report feeling unable to express themselves over webcam with limited nonverbal communication, while providers lament their lack of control over remote session settings (Almathami et al., 2020; Steidtmann et al., 2020; Uscher-Pines et al., 2020). Aspects of in-person care that are lost over TMH may be restored and enhanced with VR. VR often utilizes head-mounted displays and real-time motion tracking to create experiences of telepresence, during which users feel that simulated environments are real and distant people are here (Riva et al., 2004; Hilty et al., 2020). Telepresence facilitates gestures and body language like in-person interactions while also isolating users’ visual and auditory experience to the virtual environment. The immersive characteristics of VR make it an ideal solution to some common barriers of standard TMH (Boydstun et al., 2021; Di Carlo et al., 2021; Matamala-Gomez et al., 2021; Pimentel et al., 2021; Sampaio et al., 2021).

Accessibility of VRET is especially important due to stigmas surrounding phobias and anxiety. VRET sessions are commonly conducted in the clinical settings of a therapist’s office (Boeldt et al., 2019), which requires therapists to dedicate clinic space to VR arrangements and maintains travel burdens upon patients. It is common for people with phobias to avoid treatment and hide their symptoms, even from their own health care providers (Davidson, 2005; de Oliveira-Souza, 2018; Curcio and Corboy, 2020). Patients seeking phobia treatment often worry about embarrassing themselves in front of therapists (Bush, 2008). In one VRET study, patients in VR were so distracted by the physical presence of others that therapists had to be moved to a separate room (Jang et al., 2002). While clinic-based VRET reduces potential embarrassment for patients with immersion into simulated environments, VRET + TMH stands to extend treatment to the comfort and privacy of patients’ homes (Rizzo and Koenig, 2017).

The integration of VRET into TMH creates new options in the treatment of phobias and anxiety. The sensors in VR headsets and software can automate data collection such as patient distance or time spent touching the phobic stimuli, allowing providers to monitor patient progress remotely (Brookes et al., 2020; Krohn et al., 2020; Ratcliffe et al., 2021). Provider access to such data could facilitate patient engagement with VRET on their own time and at their own pace. Randomized trials have demonstrated successful phobia treatment with asynchronous, self-led, low-cost VRET with no negative side effects (Freeman et al., 2018; Lindner et al., 2019a; Donker et al., 2019; Shin et al., 2021). The telemonitoring of asynchronous VRET may be particularly beneficial for populations at risk for untreated phobias such as children, people who experience difficulty leaving their home, or those with limited access to in-person alternatives (Demers et al., 2020).

Scalability is another exciting prospect of VRET + TMH. There are far more patients in need than providers available to serve them, which creates constant demand for clinician force multipliers (Gardner et al., 2020). Provider-supervised digital interventions can streamline treatment delivery and be as effective as standard clinical care (Fairburn and Patel, 2017). For VRET, TMH delivery models can expand providers’ treatment options with remote asynchronous monitoring and reach patients beyond the proximity of their clinical offices (Paping et al., 2010). The convenience of automated, self-led, remotely monitored VRET can be impactful for those with subclinical phobia symptoms who may be unlikely to seek treatment (Fehm et al., 2008). Implementation of VRET + TMH could expand a provider’s capacity from one-on-one clinical sessions to mass, simultaneous care in the general population. These potential benefits of clinical scale may also extend the conduct of unsupervised VR research (Persky, 2020; Mottelson et al., 2021).

VRET + TMH may enhance collaborative session interactions. In IVET, imaginal exposure, and clinic based VRET, providers observe and communicate with a patient undergoing programmed phobic experiences. Synchronous VRET, however, allows the patient and provider to inhabit the same experiential space. Co-immersion in the VRET experience allows the provider to see what the patient sees, adjust the virtual environment in real-time response to the patient’s reactions, and interact with the phobic situation in tandem with the patient (Tabbaa et al., 2020). A user’s digital body in the virtual environment (i.e., avatar) is another empathetic aspect of VRET + TMH with important implications for mental health care (Rehm et al., 2016). Providers’ avatars can be shaped to facilitate rapport, patient comfort, and treatment procedures. In a study of VR-based therapy for eating disorders, patients felt trusting and engaged with treatment because therapists were depicted as cartoonish avatars, which patients characterized as cute, non-judgmental, and conducive to a relaxed atmosphere (Matsangidou et al., 2020). Clinic-based VRET immerses the patient into a therapeutic simulation while the therapist supervises from the outside. VRET + TMH may bring therapists into the treatment experience with patients.

TMH and VRET are clinical support tools that have proven effective in the treatment of phobias, anxiety, and mental health. Combining TMH and VRET may lead to additive benefits for evidence-based treatment since VR has been shown to enrich remote interactions and TMH can improve the deployment of VRET. While VRET + TMH can enhance respective clinical toolsets and lead to benefits neither could achieve independently, TMH and VRET each bring their own challenges. It is important to take a realistic view of critical barriers and limitations to realize the potential future of VRET delivered via TMH.

## BARRIERS TO VRET + TMH

Usability has been a concern for clinically oriented VR. It is common for therapeutic VR software to follow design conventions of video games (Tao et al., 2021). This can make VR-based therapy feel disorienting, as people who provide and receive mental health care may not have extensive gaming experience (Tuena et al., 2020; Pimentel et al., 2021). A systematic review found 1,785 VR apps for mental health, but only 11 were rated as potentially beneficial for therapy due to poor functionality (Best et al., 2021). In addition to unintuitive design or lack of therapeutic customization, VR-related motion sickness (often called cybersickness or simulator sickness) remains an issue (Timothy Zhang et al., 2020). Simulator sickness is rare in VRET research, but its occurrence can compromise patient experiences and lead to treatment dropout (Benbow and Anderson, 2019). Self-led digital interventions for mental health have notoriously high dropout rates (Saad et al., 2021), which makes usability a primary emphasis of VRET + TMH. Limiting time spent in VR to 1 h or less, brief breaks from VR within sessions, and obscuring visual motion indicators help reduce the occurrence and severity of simulator sickness (Chang et al., 2020; Kim et al., 2021). VR research continues to reveal contributing factors and techniques to minimize simulator sickness. Directly involving stakeholders in the development of their own interventions will be a vital practice to ensure optimal engagement, adoption, and ergonomics of remote VR-based therapies (Pizzoli et al., 2019; Tuena et al., 2020).

Safety is critical with emerging patient care technologies. It is unclear what design features, clinical practices, or level of immersion can optimize VRET experiences and outcomes (Botella et al., 2017; Eshuis et al., 2020). VRET allows providers to manipulate patients’ cognitive and affective states with illusory experiences, which has equal potential for positive and negative impacts. Detachment from one’s identity, body, or surroundings in VR can intensify emotional reactions and has been compared to inducing a temporary dissociative disorder (Parsons, 2021). The negative impacts of VR remain understudied for children and other populations sensitive to change in affect (Kothgassner and Felnhofer, 2021). While a meta-analysis found no substantial evidence of deleterious effects in VR therapies (Fernández-Álvarez et al., 2019), disparate study designs and publication biases may hinder detection of VR side effects (Chesham et al., 2018; Anderson and Molloy, 2020). It will be vital to promote measurement and reporting of VR side effects in research and development (Saredakis et al., 2020).

Special effort must be taken to build rapport and maintain therapeutic relationships over TMH (Glass and Bickler, 2021). While VR can restore nonverbal communication of body posture, head position, and hand gestures, VR does not yet track gaze or facial expressions. The expressionless eyes and faces of virtual characters have been hypothesized to explain weak effects in some VRET research (Kampmann et al., 2016). Facial expressions are vital data for providers to monitor during exposure therapy, but VR headsets do not track or display these data for provider assessment. Developers have acted on this same need for face and gaze tracking in commercial VR applications. HTC released a VR headset attachment to track cheek, mouth, and chin expressions (Stein, 2021). Facebook published internal research of a prototype VR headset that tracks and displays eye movements (Matsuda et al., 2021). Evidence of face and eye tracking features were also found in upcoming versions of Oculus headsets (Heaney, 2021). These are encouraging signals of forthcoming solutions to display face and gaze direction in VR-based therapies, which should be evaluated rapidly for clinical use.

Technical infrastructures are another barrier to VRET + TMH. About 27% of Americans still do not have access to affordable or reliable broadband internet (Atske, 2019). Less than half of Americans with broadband access choose to use it (McKinley, 2020). Streaming VR of acceptable quality can be achieved at 100mbps but high-end VR can require 400 Mbps of bandwidth (Mangiante et al., 2017), more than twice the average internet speed in the US (Supan, 2021). Access to VR equipment has also been complicated by COVID-19’s impacts on global supply chains and cryptocurrency mining (Molloy, 2021; The Economist, 2021). Digital divides exclude people on the basis of geography, ethnicity, gender, and socioeconomic status (Saeed and Masters, 2021). Clinicians, researchers, developers, and consumers will need to collaborate openly to avoid widening these divides in pursuit of VRET + TMH (Logan et al., 2021).

Investment and maintenance costs can be deterrents to otherwise effective health care technologies. The tendency for telemedicine to reduce costs for both patients and providers is a leading reason for its success (Almathami et al., 2020). Patients and providers may hesitate with these technologies if the potential financial gains are not apparent. Despite the falling cost of VR equipment, some mental health providers worry that VR can still be too costly (Pimentel et al., 2021). For example, investment in formal training and dedicated staff may be necessary for providers to use VR with confidence (Maples-Keller et al., 2017; Boeldt et al., 2019). However, people are willing to pay if the ends justify the means. A systematic review found that mental health providers are accepting of telemedicine costs if they believe the technology will improve patient outcomes (Harst et al., 2019). Understanding the additive, clinical value of VRET + TMH will be an important area of future research.

## DISCUSSION

Delivery of VRET as part of TMH represents a unique opportunity for cross-pollination between clinical practice, academic research, industry development, and consumer advocacy.

There can be an overwhelming number of choices in VRET + TMH. Considerations must be made for the look, feel, and sound of the VR environment and its contents; screen resolution, refresh rates, control schemes, power supply, sanitation protocols, and device maintenance; user interfaces, therapeutic content, and how all this should be different between providers and patients. It is important to disseminate how and why decisions are made for VRET + TMH to build accumulative and progress-oriented knowledge (Birckhead et al., 2019). The design of VRET, as with other healthcare technologies, is optimized most reliably through iterative collaboration with direct stakeholders (Tabbaa et al., 2020). Numerous case studies and design reports have described user-centered development processes for self-led VRET (North et al., 1997; Hartanto et al., 2016; Bălan et al., 2020). Others have reported on remote VRET user interfaces co-designed with mental health providers and patients (Brinkman et al., 2010; Kornarakis, 2017; Salkevicius and Navickas, 2018; Šalkevičius et al., 2019). Efforts to scale remote VRET have also been published on a remote VRET platform for one provider to treat multiple patients, and a system for automated tailoring of VRET exercises (Paping et al., 2010; Heyse et al., 2018). User-centered research is critical to develop a systematic understanding of VRET + TMH (Rizzo et al., 2018).

While the future is promising for VRET + TMH, there remains uncertainty about how acceptability and outcomes may vary by treatment populations, practitioner competencies, use cases, and ethics. Studies to date have not focused on diverse participant characteristics that may be relevant to risks and outcomes (Liu et al., 2020; Kaimara et al., 2021; Kothgassner and Felnhofer, 2021). TMH and VRET are not yet standard topics in professional education and training, which leaves providers with lingering hesitation (Cowan et al., 2019; Kourtesis et al., 2019; Hilty et al., 2020). Privacy regulations, practice ethics, and conceptual understandings of VR have not kept pace with the speed of technology advancements (Rizzo et al., 2018; Parker et al., 2019; Marloth et al., 2020; Slater et al., 2020). Principles and recommendations have been published for telebehavioral health technologies in clinical care and training (Maheu et al., 2018; Nazeha et al., 2020; Baier and Danzo, 2021). Such principles should be applied and revisited frequently to refine our understanding and guide the application of VRET + TMH.

As VR and telemedicine technologies continue to mature, practicality will be a leading topic for VRET + TMH. This research is expected to grow due to telemedicine’s recent uptake, but previous studies of VRET without TMH show promising cost-effectiveness. VRET for military PTSD saved $114,000 USD per patient compared to training a replacement soldier (Wood et al., 2009). Researchers in another study used only $5 USD of VR equipment, combined with off-the-shelf apps and participants’ personal smartphones, for single-session treatment of public speaking phobia (Lindner et al., 2019a). A systematic review concluded that computer simulated VRET can reduce costs compared to staging physical experiences for IVET (Botella et al., 2017), which shows promise for self-led VRET (Reeves et al., 2021). Interdisciplinary collaboration has been recommended and appears to be on the rise with a growing number of open-source VR projects (Boeldt et al., 2019; Geraets et al., 2021). Formal training and protocol manualization will also be necessary for the dissemination of VRET + TMH. A systematic review found that 1 h sessions, once weekly, for 8–12 weeks, using immersive head-mounted VR were helpful practices for VRET implementation (Krzystanek et al., 2021). Recommendations have also been made for staging the patient’s surroundings during VRET with cool temperatures and dim lighting to maximize presence and minimize distractions (Bush, 2008). Collaboration between research, health, VR, and patient groups will be necessary to expand the practical knowledge base for VRET + TMH.

There is a growing variety of off-the-shelf VR solutions that may be used to minimize development challenges and provide clinical benefit. Instead of designing and maintaining computer-generated visuals for VRET, researchers have used consumer camera systems (e.g., GoPro) to make 360 VR videos (Stupar-Rutenfrans et al., 2017; Ionescu et al., 2021). This technique facilitates production of predictable exposure hierarchies with photorealistic visuals and low production costs (Holmberg et al., 2020). Production barriers to VRET + TMH may be further reduced with clinical use of entertainment video games (Pallavicini et al., 2021). Since the early 2000s, people have sought off-label health benefits playing a virtual social game called Second Life (Beard et al., 2009). Researchers were quick to engage Second Life as a platform for health studies and education (Boulos et al., 2007), and notably for exposure-based therapy (Gorini et al., 2008). There is evidence people are using games like VRChat in a similar fashion today; as approximations of self-led VRET. Coping with social anxieties was a leading reason people started to play VRChat during the COVID-19 pandemic (Kelley, 2021; Sykownik et al., 2021). Social VR games can facilitate casual intimacy (Maloney and Freeman, 2020), which may provide opportunity for patients to engage with social phobias in low-stake settings. While most mental health VR apps have been rated unlikely to support patient care (Best et al., 2021), there is a growing abundance of other VR apps that may be useful in phobia and anxiety treatment protocols (Lindner et al., 2017). These commercial hardware, video games, and smartphone apps were not made for health purposes but there is ample opportunity to incorporate them into therapy today (Tuerk et al., 2019). Exploration of these commercial options can provide immediate clinical options and guide development of dedicated VRET + TMH solutions (Riva et al., 2020).

## CONCLUSION

Anyone can encounter experiences that inflict a phobia or anxiety disorder. These disorders, if untreated, can cascade into worsening symptoms and decline in quality of life. While in-person IVET is the current gold standard treatment for phobias, clinic-based VRET has emerged as an equally effective and more flexible treatment option. Recent advances in consumer-oriented VR hardware, growing popularity of VR entertainment, and the rapid shift to remote health care create ideal conditions to accelerate research and development of VRET + TMH. The union of TMH and VRET will be essential to meet mental health needs of the ongoing pandemic and beyond NIMH Specific Phobia, 2022.
